# Atrial Fibrillation and Chronic Kidney Disease—A Risky Combination for Post-Contrast Acute Kidney Injury

**DOI:** 10.3390/jcm10184140

**Published:** 2021-09-14

**Authors:** Łukasz Kuźma, Anna Tomaszuk-Kazberuk, Anna Kurasz, Małgorzata Zalewska-Adamiec, Hanna Bachórzewska-Gajewska, Sławomir Dobrzycki, Marlena Kwiatkowska, Jolanta Małyszko

**Affiliations:** 1 Department of Invasive Cardiology, Medical University of Bialystok, 24A Sklodowskiej-Curie St., 15276 Bialystok, Poland; kuzma.lukasz@gmail.com (Ł.K.); annaxkurasz@gmail.com (A.K.); mzalewska5@wp.pl (M.Z.-A.); hgajewska@op.pl (H.B.-G.); slawek_dobrzycki@yahoo.com (S.D.); 2 Department of Cardiology, Medical University of Bialystok, 24A Sklodowskiej-Curie St., 15276 Bialystok, Poland; a.tomaszuk@poczta.fm; 3 Department of Clinical Medicine, Medical University of Bialystok, 24A Sklodowskiej-Curie St., 15276 Bialystok, Poland; 4 Department of Nephrology, Dialysis and Internal Disease, Medical University of Warsaw, 1A Banacha St., 02097 Warsaw, Poland; marlenakwiatko@gmail.com

**Keywords:** atrial fibrillation, post-contrast acute kidney injury, acute kidney injury, chronic kidney disease, coronary artery disease

## Abstract

Atrial fibrillation (AF) symptoms may mimic coronary artery disease (CAD) which reflects the difficulties in qualifying AF patients for invasive diagnostics. A substantial number of coronary angiographies may be unnecessary or even put patients at risk of post-contrast acute kidney injury (PC-AKI), especially patients with chronic kidney disease (CKD). We aimed to investigate the hypothesis indicating higher prevalence of PC-AKI in patients with AF scheduled for coronary angiography. The study population comprised of 8026 patients referred for elective coronarography including 1621 with AF. In the comparison of prevalence of PC-AKI in distinguished groups we can see that kidney impairment was twice more frequent in patients with AF in both groups with CKD (CKD (+)/AF (+) 6.24% vs. CKD (+)/AF (−) 3.04%) and without CKD (CKD (−)/AF (+) 2.32% vs. CKD (−)/AF (−) 1.22%). In our study, post-contrast acute kidney disease is twice more frequent in patients with AF, especially in subgroup with chronic kidney disease scheduled for coronary angiography. Additionally, having in mind results of previous studies stating that AF is associated with non-obstructive coronary lesions on angiography, patients with AF and CKD may be unnecessarily exposed to contrast agent and possible complications.

## 1. Introduction

Atrial fibrillation (AF) is the most commonly occurring arrhythmia, while coronary artery disease (CAD) is the most common cardiovascular disease and remains the leading cause of death worldwide [1,2]. Both conditions share several common risk factors—smoking, obesity, diabetes mellitus, obstructive sleep apnea, and elevated blood pressure. Moreover, some of the symptoms overlap, thus AF manifestation can mimic coronary artery disease [3,4,5,6,7]. This poses difficulties in qualifying AF patients for invasive diagnostics, given that atrial fibrillation is associated with the lack of significant coronary lesions on angiography [8].

The chronic kidney disease (CKD) has in common some of the above-mentioned risk factors and its co-occurrence with AF is increasingly prevalent in the general population [9]. CKD is also associated with increased frequency of post-contrast acute kidney injury (PC-AKI) after coronary angiography. The pathophysiology of PC-AKI is not yet understood precisely, therefore new studies exploring the subject, as well as preventive methods, should be pursued continuously [10,11].

**Figure 1:**
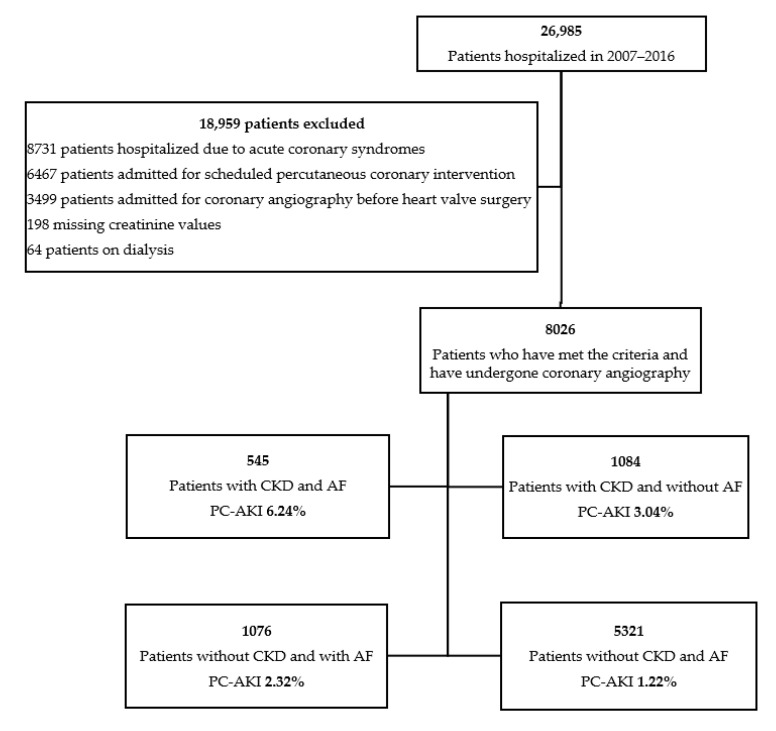
Selection of the study population.

A substantial number of coronary angiographies may be unnecessary or even put the patient at risk of post-contrast acute kidney injury, especially patients with chronic kidney disease (CKD). Therefore, our goal was to investigate the hypothesis indicating higher prevalence of post-contrast acute kidney failure in patients with AF scheduled for coronary angiography. As shown in Figure 1, the cohort of participants was divided into four groups, as follows: CKD (+)/AF (+), CKD (+)/AF (−); CKD (−)/AF (+); CKD (−)/AF (−).

## 2. Materials and Methods

We reviewed medical records of 26,985 patients hospitalized in the Department of Invasive Cardiology of the Medical University of Bialystok (Bialystok, Poland) for coronary angiography between 2007 and 2016. We excluded patients with chronic coronary syndromes (CCS), acute coronary syndromes (ACS), and those referred for scheduled percutaneous coronary intervention (PCI) or angiography before heart valve surgery. Dialysis and missing creatinine values were also the exclusion criteria (Figure 1).

Eventually, our final study cohort consisted of 8026 patients. All patients have undergone coronary angiography. During procedures an iodine-containing non-ionic radiocontrast agent was used. All patients had the same strategy for prevention of radiocontrast agent. Coronary angiography was performed according to the Judkins technique [12]. Diagnosis of CCS and indication for PCI were performed according to current ESC guidelines [13]. Significant stenosis of the coronary vessel was defined as more than 50% in main stem of the left coronary artery and 70% in the rest of the vessels. CCS degree was classified as single-, double- or multi-vessel disease.

CKD-EPI eGFR and creatinine levels were assessed on admission. The term PC-AKI has been used according to the distinction based on the recommendations, which determines the use of the term contrast-induced acute kidney injury (CI-AKI) only when we can pinpoint a causal connection between the administration of contrast media and acute renal impairment as opposed to PC-AKI [14]. For the purpose of this study, PC-AKI was defined as an absolute increase of serum creatinine ≥0.5 mg/dL or a relative increase ≥25% from baseline value within the first 48–72 h of intervention [15]. All patients had the same strategy for the prevention of radiocontrast agent—1000 mL of intravenous hydration with 0.9% NaCl as well as withdrawal of Metformin before the procedure regardless of eGFR value [16]. As for the volume of contrast media each patient is given a particular volume according to the procedure specifications: 40 mL for a diagnostic coronarography, 55 mL for a diagnostic coronarography with left ventricular angiography. Volume of contrast media for a diagnostic catheterization and PCI varies depending on complexity of the procedure, e.g., the number of stents.

The subgroup of patients with atrial fibrillation was defined as the presence of AF on an electrocardiogram during the index hospitalization and/or as indicated by a diagnosis found in medical records. Diagnoses and AF classification were based on physician-assigned diagnoses in the medical records and/or the presence of corresponding ICD-10 codes [17]. Less than 1% of the data were missing and these data were excluded from the analysis.

### Statistical Analysis

Distribution of the variables was assessed with the Kolmogorov-Smirnov test. Data are expressed as means and standard deviations (SD). Relative frequencies are used to present categorical variables.

Statistical significance of differences between PC-AKI and non PC–AKI patients were determined using the Student’s T-test and Mann-Whitney test.

To compare four groups for non-normal distributed variables we used Kruskal-Wallis test with multiple pairwise comparisons using the Steel-Dwass-Critchlow-Fligner procedure, for categorical variable χ^2^ test was used.

Multivariable backward stepwise selection logistic regression was used to determine the odds ratio of having post-contrast acute kidney injury. Model included all predictors with a *p* value of less than 0.1 and without a significant multicollinearity effect. The variance inflation factor was used to identify a correlation—and the strength of that correlation—between independent variables. The data are presented as odds ratios with 95% confidence intervals.

The *p*-value < 0.05 was considered statistically significant. The statistic software Microsoft Excel (Microsoft, version 16.40, Redmond, WA, USA, 2020) and XL Stat (Addinsoft, version 2020.03.01, New York, NY, USA, 2020) were used.

## 3. Results

A total of 8026 patients were qualified for the study, of which more than a half were men (54.06%) with a mean age of 65.26 years (SD = 10.14) (Figure 1). Separate analyses were carried out, distinguishing patients depending on presence or absence of AF and CKD.

The male gender was dominant in PC-AKI patients (70.06% (N = 110) vs. 53.74 (N = 4229), *p* < 0.001). They more often had atrial fibrillation (37.58% (N = 59) vs. 19.85 (N = 1562), *p* < 0.001), chronic kidney disease (42.68% (N = 67) vs. 19.85 (N = 1562), *p* < 0.001), lower mean ejection fraction (41.53 (SD = 16.97) vs. 50.43 (SD = 13.29), *p* < 0.001), and were also more likely to have significant stenosis (58.6% (N = 92) vs. 39.94 (N = 3144), *p* < 0.001). In addition, patients with PC-AKI were more often treated with NOAC (*p* = 0.03) and VKA (*p* = 0.008) anticoagulants (Table 1).

There were substantial differences in terms of clinical characteristics between groups with and without AF and CKD, details are presented in Table 2. Comparing two CKD (+) subgroups the one with AF was twice more likely to have a PC-AKI than the one without AF (6.24% (N = 34) vs. 3.04% (N = 33), *p* < 0.001). CKD (−)/AF (+) group more often than CKD (−)/AF (−) one had significant stenosis (33.55% (N = 361) vs. 39.3% (N = 2091), *p* < 0.001). CKD (+)/AF (+) subgroup had the highest concentration of fibrinogen as well as serum creatinine (*p* < 0.001). Patients without CKD and AF had the highest value of eGFR (*p* < 0.001).

**Figure 2:**
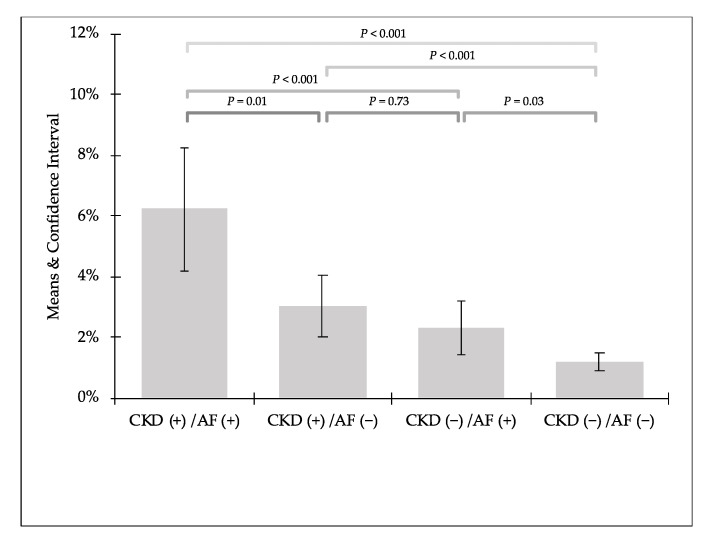
Detailed comparison of prevalence of post-contrast acute kidney injury.

In the comparison of prevalence of PC-AKI in distinguished groups we can see that kidney impairment was twice more frequent in patients with AF in both groups with CKD (CKD (+)/AF (+) 6.24% vs. CKD (+)/AF (−) 3.04%) and without CKD (CKD (−)/AF (+) 2.32% vs. CKD (−)/AF (−) 1.22%) (Figure 1 and Figure 2).

**Figure 3:**
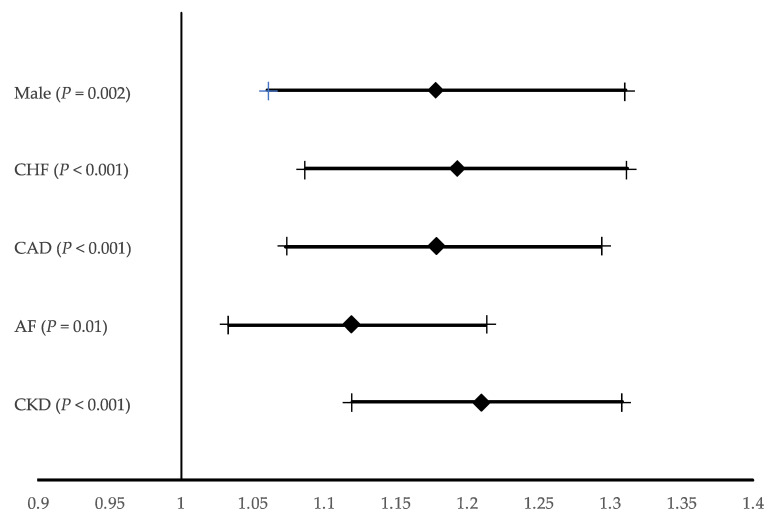
Multivariable backward stepwise selection logistic regression model. Odds ratio for post-contrast acute kidney injury. The data are presented as odds ratios with 95% confidence intervals (Hosmer-Lemeshow Statistic 0.599). AF, atrial fibrillation; CAD; Coronary artery disease; CHF, Chronic Heart Failure; CKD, chronic kidney disease.

The risk of PC-AKI increased with occurrence of CKD (OR 1.210; 95% CI 1.120–1.308; *p* < 0.001), atrial fibrillation (OR 1.120; 95% CI: 1.033–1.214; *p* = 0.006) and CHF (OR 1.194; 95% CI: 1.087–1.312; *p* < 0.001) and coronary artery disease (OR 1.178; 95% CI: 1.073–1.294; *p* < 0.001). These results were also dependent on sex (OR for males 1.790; 95% CI 1.061–1.311; *p* < 0.001) (Figure 3).

## 4. Discussion

The incidence of atrial fibrillation has increased over the last years and will continue to increase in the coming ones, giving us an estimated 17.9 million cases in Europe by 2060 [4,18]. There are a number of factors that determine the origin of AF from hypertension, valvular defects, diabetes, hyperthyroidism to heart failure and coronary artery disease [19,20].

While the occurrence of AF among patients with coronary artery disease is rather low, reaching up to 5%, the prevalence of CAD among patients with AF can reach higher values [21,22]. In our study, CAD was presented in 37.51% of patients with atrial fibrillation, while in patients with sinus rhythm was 41.01%. According to other research, this number varies from 17% to even 46.5% [23,24,25,26].

Due to intensified symptoms in atrial fibrillation, which are reflected in the EHRA scale, patients at an early stage of the disease are referred to invasive diagnostics—coronary angiography, since symptoms resembling CAD manifest themselves [27]. Traditional stress tests, as one of the available non-invasive CAD diagnostic methods, may be non-conclusive in patients with AF. Pradhan et al. in their study observed that ST-segment depression during rapid AF is not predictive for the presence of obstructive coronary artery disease [28]. Single-photon emission computed tomography (SPECT) exercise test also showed limited accuracy in this group of patients [29]. A good alternative can be dobutamine stress echocardiography, even in patients with AF it still has high accuracy to uncover CAD [30], but physicians refer their patients for these tests unwillingly because they are aware of the possibility of evoking AF episode in patients with paroxysmal AF. Additionally, CT scan requires a slow heart rate, which is hardly possible in patients with AF. Therefore, having in mind all the limitations of other diagnostic alternatives, coronary angiography remains the preferred option, despite possible complications.

One of the possible complications after coronary angiography is post-contrast acute kidney injury (PC-AKI) and it is known that patients with CKD have a higher risk of this adverse outcome [31]. In addition to this connection, the occurrence of PC-AKI may also lead to the development of chronic kidney disease as well as herald adverse outcomes in patients undergoing percutaneous coronary intervention due to acute coronary syndrome [7,32]. Regarding the adverse long-term impact of PC-AKI, opinions are disputed due to the lack of direct causation in the studies, however, increased mortality, a progression of chronic kidney disease, and kidney failure were observed [11]. The factors contributing to the development of acute kidney injury after contrast, beside CKD, are older age, hemodynamic instability, congestive heart failure, diabetes mellitus, anemia, and the volume of contrast media [10,33]. Moreover, markers are being sought that will allow for early detection of PC-AKI and implementation of appropriate treatment strategies that might improve clinical outcomes. The latest meta-analysis by Li et al. suggests effective predictive value of BNP or NT-proBNP in acute kidney impairment identification [34]. A study by Chen et al. revealed that long non-coding RNAs -HILPDA and -PRND may serve as the novel biomarkers in this area with 100% sensitivity and 83.93% specificity [35].

There are several large population studies that consistently show a higher prevalence of AF among patients with CKD than in those without kidney disease [36]. Taking this into account, it can be assumed that patients with this set of comorbidities may belong to a high-risk group of post-contrast acute kidney injury. This is reflected in our analysis, PC-AKI was directly related with occurrence of AF, CKD, CAD, chronic heart failure, and male sex. Considering these results, it is also important to take appropriate preventive measures among patients who will require coronarography to avoid kidney damage [37,38].

Possible underlying mechanisms that may contribute to development of contrast-induced nephropathy in patients with atrial fibrillation may include acute hemodynamic changes at the time of procedure due to sedation, transient atrial stunning, other severe comorbidities, and hypovolemia from fasting state may result in renal hypoperfusion [39,40]. Mechanical atrial stunning might be implicated in a lack of improvement of cardiac output immediately after PCI and may also be a contributing factor due to a decrease in renal perfusion [41]. In addition, the volume status should be optimized before the PCI with judicious use of diuretics to avoid hypovolemia or better withdrawal of diuretics if possible. The presence of CKD is a known risk factor for AKI; however, this relationship is difficult to assess due to the presence of many confounders [42]. Micro-thromboembolism has been postulated to play a role in CKD and cognitive decline [43]. It may also be relevant for AKI. In the recent study incidence and the risk factors of in-hospitalized AKI patients hospitalized for AF were published by Wang et al. [44]. They found that detection rate of in-hospital AKI in Chinese patients with AF was 8.0%. The risk factors for in-hospital AKI in this population were age (per 10-year increase), the use of diuretics before admission, and baseline hemoglobin (per 20 g/L decrease). Moreover, Harel et al. [45] in a very recent population-based cohort study of 20,683 outpatients in Ontario (Canada) assessed the risk of AKI among elderly individuals (more than 66 years old) with atrial fibrillation newly prescribed a DOAC (dabigatran, rivaroxaban, or apixaban) versus warfarin. They postulated several mechanisms for anticoagulant-induced AKI such as: systemic bleeding leading to hypotension, acute interstitial nephritis, and anticoagulant related nephropathy, a condition mediated by glomerular hemorrhage leading to the formation of obstructive red cell casts in distal tubules and free radical injury from lysed red blood cells particularly at supratherapeutic level of anticoagulation [46,47]. However, due to the retrospective analysis, we were not able to establish causative relations between AKI/CKD and treatment of AF. In the elegant review Brodsky and Hebert [48] discussed anticoagulant-related nephropathy. They stressed that it occurred mainly in those who already had multiple risk factors for AKI (e.g., CKD, cardiovascular disease, and diabetes mellitus), i.e., AKI is multifactorial. In addition, diagnosis is of exclusion, unless a kidney biopsy is performed and nephrologists are naturally reluctant to consider kidney biopsy in anticoagulated patients due to higher risk of bleeding. These potential mechanisms are merely speculative based on observational data and require additional studies. Similar possible mechanisms except contrast-agent may underlie the worsening of kidney function following direct current cardioversion [49].

Overall, coronary angiography is the most effective method used to evaluate coronary vessels, especially in patients with acute coronary syndrome. In one of our previous studies, we observed that a substantial number of patients with atrial fibrillation show no significant narrowing of coronary arteries during coronarography [50]. In the present analysis, 62.49% of patients with AF presented with non-obstructive coronary lesions on angiography. Given how many symptoms of coronary artery disease and atrial fibrillation overlap, it is crucial to use all non-invasive methods to differentiate them. This way proportion of non-significant coronary angiographies in patients with atrial fibrillation will decrease, and therefore the amount of PC-AKI will be reduced.

## 5. Conclusions

In our study post-contrast acute kidney disease is twice more frequent in patients with AF, especially in subgroup with chronic kidney disease scheduled for coronary angiography. Additionally, having in mind results of previous studies stating that AF is associated with non-obstructive coronary lesions on angiography, patients with AF and CKD may be unnecessarily exposed to contrast agent and possible complications.

## Figures and Tables

**Table 1:** Characteristics of the studied population with comparison of patients with and without post-contrast acute kidney injury.

	All Study Participants (N = 8026)	Patients with PC–AKI (N = 157)	Patients without PC-AKI (N = 7869)	*p*
Age (years); mean (SD)	65.26 (10.14)	67.6 (11.06)	65.21 (10.12)	0.003
Male; % (N)	54.06 (4339)	70.06 (110)	53.74 (4229)	<0.001
BMI (kg/m^2^); mean (SD)	29.13 (4.85)	29.36 (5.39)	29.12 (4.84)	0.57
Atrial fibrillation; % (N)	20.2 (1621)	37.58 (59)	19.85 (1562)	<0.001
Chronic kidney disease; % (N)	20.3 (1629)	42.68 (67)	19.85 (1562)	<0.001
Patients with eGFR ≥90 mL/min/1.73 m²; % (N)	26.15 (2099)	21.02 (33)	26.25 (2066)	0.14
Patients with eGFR 60–89 mL/min/1.73 m²; % (N)	55.58 (4461)	43.95 (69)	55.81 (4392)	0.003
Patients with eGFR 45–59 mL/min/1.73 m²; % (N)	12.14 (974)	14.01 (22)	12.1 (952)	0.47
Patients with eGFR 30–44 mL/min/1.73 m²; % (N)	5.12 (411)	16.56 (26)	4.89 (385)	<0.001
Patients with eGFR 15–29 mL/min/1.73 m²; % (N)	0.96 (77)	4.46 (7)	0.89 (70)	<0.001
Patients with eGFR <15 mL/min/1.73 m²; % (N)	0.05 (4)	0 (0)	0.05 (4)	0.78
Serum creatinine concentration (mg/dL); mean (SD)	0.95 (0.27)	1.14 (0.45)	0.95 (0.26)	<0.001
CKD-EPI eGFR mL/min/1.73 m²; % (N)	76.85 (18.21)	68.67 (22.92)	77.01 (18.06)	<0.001
Chronic heart failure; % (N)	32.22 (2586)	41.53 (16.97)	50.43 (13.29)	<0.001
Ejection Fraction; mean (SD)	50.22 (13.45)	41.53 (16.97)	50.43 (13.29)	<0.001
Hypertension; % (N)	83.17 (6675)	82.17 (129)	83.19 (6546)	0.74
Diabetes mellitus; % (N)	25.65 (2059)	30.57 (48)	25.56 (2011)	0.15
Hyperlipidemia; % (N)	67.46 (5414)	61.78 (97)	67.57 (5317)	0.13
Significant stenosis; % (N)	40.31 (3235)	58.6 (92)	39.94 (3143)	<0.001
Single-vessel CAD; % (N)	18.1 (1453)	28.03 (44)	17.91 (1409)	0.009
Double-vessel CAD; % (N)	9.68 (777)	8.28 (13)	9.71 (764)	0.549
Multi-vessel stenosis; % (N)	22.2 (1782)	30.57 (48)	22.04 (1734)	0.02
Hemoglobin level (g/dL); mean (SD)	13.71 (1.38)	13.42 (1.65)	13.71 (1.37)	0.01
RBC (mln/mm^3^); mean (SD)	4.58 (0.47)	4.51 (0.56)	4.58 (0.47)	0.048
Hematocrit (%); mean (SD)	40.83 (3.86)	40.27 (4.19)	40.84 (3.85)	0.07
Platelets account (×10^3^/mm^3^); mean. (SD)	225.48 (69.71)	212.97 (61.36)	225.7 (69.83)	0.04
Serum potassium concentration (mEq/L); mean (SD)	4.43 (0.43)	4.52 (0.58)	4.43 (0.42)	0.02
Serum LDL cholesterol concentration (mg/dL); mean (SD)	105.14 (40.05)	105.53 (37.35)	105.13 (40.1)	0.91
Serum HDL cholesterol concentration (mg/dL); mean (SD)	47.83 (13.23)	45.04 (13.78)	47.88 (13.21)	0.01
Fibrinogen concentration (mg/dL); mean (SD)	389.54 (86.95)	411.45 (86.63)	389.1 (86.91)	0.002
NOAC anticoagulants prescribed at discharge; % (N)	4.14 (332)	7.64 (12)	4.07 (320)	0.03
VKA anticoagulants prescribed at discharge; % (N)	11.72 (941)	18.47 (29)	11.59 (912)	0.008

BMI, body mass index; CAD, coronary artery disease; CKD, chronic kidney disease; eGFR, estimated glomerular filtration rate; HDL, high density lipoprotein; LDL, low density lipoprotein; NOAC, non-vitamin K oral anticoagulants; PC–AKI, post-contrast acute kidney injury; RBC, Red blood cells; VKA; vitamin K antagonists.

**Table 2:** Comparison of patients with and without chronic kidney disease and atrial fibrillation.

	CKD (+)/AF (+) (N = 545) ^A^	CKD (+)/AF (−) (N = 1084) ^B^	CKD (−)/AF (+) (N = 1076) ^C^	CKD (−)/AF (−) (N = 5321) ^D^	*p*
PC–AKI; % (N)	6.24 (34)	3.04 (33)	2.32 (25)	1.22 (65)	<0.001 ^1^
Age (years); mean (SD)	73.16 (7.85)	71.98 (8.7)	66.31 (9.57)	62.86 (9.66)	<0.001 ^2^
Male; % (N)	49.54 (270)	44.19 (479)	66.45 (715)	54.03 (2875)	<0.001 ^3^
BMI (kg/m^2^); mean (SD)	29.89 (5.59)	28.83 (4.84)	30.01 (5.1)	28.94 (4.69)	<0.001 ^4^
Chronic heart failure; % (N)	67.71 (369)	34.59 (375)	59.01 (635)	22.68 (1207)	<0.001
Serum creatinine concentration (mg/dL); mean (SD)	1.33 (0.32)	1.28 (0.39)	0.89 (0.15)	0.86 (0.14)	<0.001
CKD-EPI eGFR ml/min/1.73 m²; % (N)	48.58 (11.66)	51 (12.03)	81.21 (12.24)	84.13 (12.31)	<0.001
Significant stenosis; % (N)	45.32 (247)	49.45 (536)	33.55 (361)	39.3 (2091)	<0.001 ^2^
Multi-vessel stenosis; % (N)	19.45 (106)	28.32 (307)	15.52 (167)	22.59 (1202)	<0.001 ^5^
Fibrinogen concentration (mg/dL); mean (SD)	421.31 (93.5)	408.18 (97.63)	395.36 (92.01)	381.29 (81.29)	<0.001
NOAC anticoagulants prescribed at discharge; % (N)	18.35 (100)	0.0 (0)	21.1 (227)	0.1 (5)	<0.001 ^4^
VKA anticoagulants prescribed at discharge; % (N)	60 (327)	0.0 (5)	55.67 (599)	0.2 (10)	<0.001 ^4^

^1^ no significant differences between the B vs. C group. ^2^ no significant differences between the A vs. B group. ^3^ no significant differences between the A vs. B and D groups. ^4^ no significant differences between the A vs. C and B vs. D groups. ^5^ no significant differences between the A vs. C and D groups. AF, atrial fibrillation; BMI, body mass index; CKD, chronic kidney disease; eGFR, estimated glomerular filtration rate; LDL, low density lipoprotein; NOAC, non-vitamin K oral anticoagulants; PC–AKI, post-contrast acute kidney injury, VKA; vitamin K antagonists.

## Data Availability

The data presented in this study are available on request from the corresponding author.
